# Effect of Freezing on the Shelf Life of Salmon

**DOI:** 10.1155/2018/1686121

**Published:** 2018-08-12

**Authors:** Paul Dawson, Wesam Al-Jeddawi, Nanne Remington

**Affiliations:** Department of Food, Nutrition and Packaging Sciences, Clemson University, Clemson, SC 29634, USA

## Abstract

Food shelf-life extension is important not only to food manufacturers, but also to home refrigeration/freezing appliance companies, whose products affect food quality and food waste. While freezing and refrigerating both extend the shelf life of foods, food quality deterioration continues regardless of the preservation method. This review article discusses the global fish market, the composition of fish meat, and the effects of freezing and thawing on salmon quality.

## 1. Introduction

Consumer preference is vital to sustaining any food commodity including the fish industry. Since 1961 international fish intake has increased as fast at 3.6% per year [1]. Whether locally caught and consumed fresh or distributed frozen, the health benefits of adding fish to the daily diet has impacted consumption [1]. Atlantic salmon (*Salmo Salar*) shows cardiovascular, cancer inhibiting, and joint health benefits mostly due to the presence of omega-3 long-chain fatty acids, eicosapentaenoic acid (EPA), and docosahexaenoic acid (DHA), some of which are essential and important nutrients for human body [2]. It is also a highly oily fish known to be low in mercury, like tuna, catfish, and cod [2]. Therefore, the quality of Atlantic salmon is important for palatability especially for frozen fish [3]. While freezing will slow the biological, chemical, and physical deterioration of food, degradation of food quality such as color, texture, enzymatic activity, lipid oxidation, and ice crystal structural damage still occur. Many researchers have reported that fast freezing results in rapid ice nucleation within the intracellular areas of food products creating smaller and more uniform ice crystals causing less structural damage to the product [4]. The denaturation of the protein is one problem caused by slow freezing with protein denaturation-dependent upon temperature [5]. Freezing at low temperatures provided small ice crystals which increased light scattering and absorption across all wavelengths in the visible region [6]. Several researchers showed that freezing even in the short term changed physical properties such as weight loss, color, and texture of the Atlantic salmon and other types of fish [7]. Long-term frozen storage leads to slow deterioration in the quality of salmon which can differ due to the storage temperature. Biological and chemical reactions such as enzymatic activity and lipid oxidation have a significant impact on fish quality during long-term frozen storage [8, 9]. Lipid oxidation decreases the sensory quality of fish and fish products and is influenced by handling and processing of fish which can also impact nutritional quality, texture, and color [10, 11].

Freezing preserves quality allowing an expanded distribution range for raw fish; thus research into different freezing methods and their effect on quality is important to the seafood industry. Quality measures affected by freezing include changes in color, texture, water holding capacity, and intracellular/extracellular ice crystal growth effects on structure. Faster freezing rates maintained structural quality and lowered chemical activity of Atlantic sea bass, Atlantic salmon, hake, chicken, and other types of meat [12–18]. However, there was a threshold-freezing rate after which an increase in freezing rate did not impact overall product quality during storage [19]. Therefore, more research is needed to find a balance between maintaining food quality, while maximizing energy efficiency of household and commercial freezers.

## 2. Global Fish Consumption

**Figure 1:**
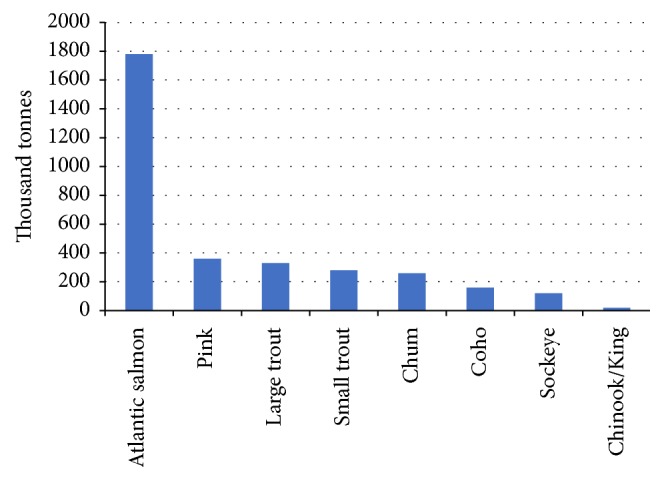
2012 Worldwide Salmonids Harvest (species).

Atlantic salmon* (Salmo salar) *is a migratory fish found widely in the northern Atlantic Ocean and adjacent freshwater. During the few past decades, it has become an important marine fish species in food markets (Figure 1).

**Figure 2:**
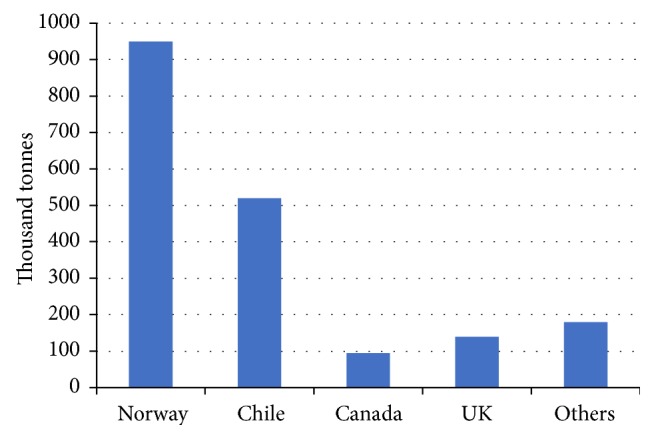
2011 Worldwide Salmonids Harvest (place of production) (created from [20]).

In 2012, the total harvest of Atlantic salmon was 1.78 million tones. As the largest species of edible salmonids, Atlantic salmon can be prepared in many ways such as smoking, grilling, and sushi. Atlantic salmon processing industry requires high quality salmon, especially for sushi consumption. Inland farming of Atlantic salmon occurs in Norway, Chile, UK, North America, and New Zealand/Tasmania (Figure 2). Recent data shows North America has become the second largest Atlantic salmon market in the world having only 37 present of entire demand fulfilled by the harvest of its own.

Salmon is harvested and frozen in Norway and South America and then transported to North America by cargo ship. Generally, freezing of Atlantic salmon is necessary according to both industrial procedures and federal legislation. However, processing and retail establishments have a high demand for fresh Atlantic salmon and the shelf-life of the fresh salmon is an important factor influencing the salmon industry.

Worldwide fish consumption per capita in 1960 and 2015 was 9.9 kg and 20.4 kg, respectively [20]. According to FAO, fish consumption will continue to increase in the coming years and is predicted to increase to 21.8 kg in 2025 which is equivalent to another 28 million tons of seafood [20]. The greatest increase in fish consumption is expected to be primarily in Asia and developing countries leading to an increase in world trade [23]. Fish consumption in developing countries is predicted to increase by 57% from 62.7 million tons in 1997 to 98.6 million tons in 2020. While in comparison, developed country fish consumption is estimated to increase only 4% from 28.1 million tons in 1997 to 29.2 million tons in 2020. The consumption of salmon in the United States varies by species, product, origin (domestic and imported), and type (wild and farmed) [24]. The average annual total US salmon consumption from 2000 to 2004 was 284,000 metric tons and was comprised of 105,000 metric tons (37%) of Pacific salmon (chinook, sockeye, coho, pink, and chum) and 180,000 metric tons (63%) of Atlantic salmon. US consumption of fresh salmon over the same period was 63%, while canned salmon was 16% and frozen salmon 21%. In addition, 68% of US salmon consumption was imported and 65% was farmed [24]. Total US salmon consumption increased dramatically from 130,000 metric tons in 1989 to more than 300,000 metric tons in 2004 [24]. Part of the reason for the increased consumption is due to perceived health benefits from regular consumption of fish which include protection against human pathogens [25], reduction of heart diseases, and risk of developing dementia, including Alzheimer's disease [26].

## 3. Proximate Composition of Fish

The proximate composition of fish is in the range of 16-21% protein, 0.2-5% fat, 1.2-1.5% mineral, 0-0.5% carbohydrate, and 66-81% moisture [27, 28]. However, fish of various species do not provide the same nutrient profile to the consumer [29] and the nutritive value of a fish varies with season [30]. The major component in fish is moisture which is a determinant of the value of the products, sensory attributes, and shelf-life in fish [31]. To reduce moisture or drip loss during frozen storage and thawing, the commercial fish production has improved the retention and addition of water to fish during harvest, processing, and storage. However, water addition to make up for moisture losses and excessive extraneous water addition for economic gain can negatively affect quality and is fraudulent [31]. Protein is the most predominant component in fish other than moisture and most finfish muscle tissue such as salmon consists of 18–22% crude protein [32]. Fish protein has a complete amino acid profile and high degree of digestibility [33]. The third and fourth major components in fish are lipid and ash. The total lipid and ash content of fish vary with the size of the fish and may also vary with the season and habitat from which the fish is harvested [34]. Fat content may vary widely due to fish species, muscle, and how a fillet is cut [35]. For example, the fat content of Norwegian salmon fillets ranged from 11% to 19% [36]. Fish oil contains a high amount of polyunsaturated fatty acids (omega-3 fatty acids) which can decrease the serum cholesterol and prevent heart diseases [37, 38]. Moreover, fish provide a great source of vitamins and minerals such as vitamins A and D, phosphorus, magnesium, selenium, and iodine [20].

## 4. Fish Muscle Structure

**Figure 3:**
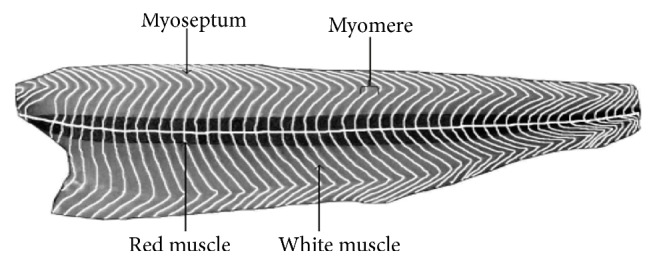
Salmon fish fillet in longitudinal section, beneath the skin, to present the W-shape of myomere and the two muscle types (Figure 3 is reproduced from [21] [under the Creative Commons Attribution License/public domain]).

**Figure 4:**
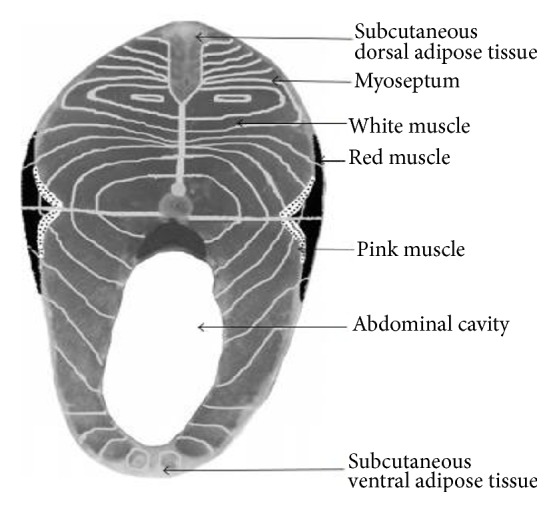
Organization and distribution of muscle mass on a trout cutlet (Figure 4 is reproduced from [21] [under the Creative Commons Attribution License/public domain]).

Fish have a distinctive muscle structure that rapidly degrades postmortem. Fish structure can vary based upon species, seasonality, maturity, and living environment but have a similar red and white muscle structure (Figures 3 and 4). Fish muscle can be structurally divided into myotomes, which are separated by connective tissue called myocomma or myoseptum [21]. There are red, white, and mosaic skeletal muscle groups, all having different functions. Red muscle represents around 30% of fish skeletal muscle and is the primary muscle type involved in highly aerobic activities, such as swimming. Red muscles contain high levels of lipids and are subsequentially subject to lipid oxidation, especially in fatty fish, such as Atlantic salmon. Trimethylamine oxides are also found within the red muscle that can be enzymatically or nonenzymatically degraded, resulting in products such as dimethylamine (DA) and formaldehyde (FA) [39, 40]. White muscle makes up around 70% of fish muscle structure and is associated with anaerobic activity. White muscle more readily reduces glycogen to lactic acid than other fiber types [41]. Finally, mosaic muscles are muscle locations in fish where mixtures of red and white muscle coexist. These areas in fish muscle are seen more frequently in fatty fish, such as Atlantic salmon. Both the lipid oxidative capabilities of fish muscle and chemical side reactions between lipids and proteins causing protein aggregation and denaturation are linked to muscle structure activity within Atlantic salmon contributing to quality change during short- and long-term storage [42].

## 5. Quality Measurements Affected by Frozen Storage

Major physical and chemical attributes that change during freezing and frozen storage in fish are color, texture, enzymatic activity, lipid oxidation, and ice crystal structural damage. Most studies on Atlantic salmon and other types of fish show physical changes during freezing (short-term effects), such as weight loss, color change, and structural/texture changes because of ice crystal nucleation and growth [6, 7]. During longer term storage, physical attributes continue to slowly deteriorate; however, chemical characteristics such as enzymatic activity, lipid oxidation, and microbial growth become increasingly important factors affecting meat quality [8, 43]. Because of the difference in quality that short- and long-term freezing effects can have on products, studying both phases of freezing is necessary in determining the effects on Atlantic salmon.

### 5.1. Weight Loss

Weight loss by ice sublimation has been widely studied [4, 17, 44–47]. Freeze and thaw weight loss for salmon decreased with faster freezing rates [4, 17]. Weight loss within frozen and thawed salmon occurred because of damaged induced by ice crystal growth during the freezing process [17]. There has not been extensive research on freeze loss specifically as a quality measure in weight loss analysis, as drip loss (thaw/cook loss) is more common. However, with ice crystal pore morphology studies, the freezing process transfers freestanding water molecules into a uniform crystal lattice unit [13]. Atlantic salmon or other fish frozen at higher freezing rates freeze more quickly and retain structural integrity in the intracellular muscle structure since more and smaller ice crystals are formed resulting in fewer freestanding or thermodynamically unstable water/ice molecules. Salmon placed in higher temperatures freezers freeze more slowly and as a result accrue larger, less uniform ice crystals. As ice crystals form in extracellular and intracellular areas around fish muscle structure, cell membrane damage causes less water to be bound within the muscle structure [13]. Thawing can further damage meat structure and the period during which the damage from slow freezing manifests itself. During thawing ice crystals melt, and if formed intracellularly or around muscle tissue, moisture would remain within the fish. However, samples with cell membrane damage cannot retain unbound water [4]. Air blast freezing caused greater drip loss in Atlantic salmon compared to faster freezing methods such as pressure shift freezing [17]. Muscle fiber shrinkage will also cause higher drip loss during freezing, which occurred in salmon samples frozen more slowly [48]. When water is frozen slowly and unbound from muscle structure, muscle fibers retract due to dehydration of concentrated proteins and minerals [49]. Unlike with pork as reported by [50], salmon freeze and thaw loss were statistically different between treatment samples during freezing/thawing.

### 5.2. Color

Color or appearance is a physical attribute that can change during freezing resulting from deterioration at the food surface, although changes in pigment appearance can be due to both chemical and biological actions. Color can affect product perception without affecting nutrition or flavor [42]. Different forms of color analysis of food products are available to predict consumer acceptance and track color changes in frozen products [51]. Fish appearance and color originate from meat-water binding properties and pigmentation within the skin or meat surface. Depending on the fish species, pigmentation can be oxidized resulting in darkening or fading. Salmon meat has a pink pigmentation in its natural state and with freezing; the pink color tends to fade [42]. Fading or increase in lightness is related to ice crystal formation during freezing [6]. Higher freezing rates form small, more numerous ice crystals within salmon, which then reflect light more intensely. Slower freezing rates form larger and fewer ice crystals in salmon, resulting in light refraction and a darkening effect of the meat surface. To analyze color changes in products the International Commission of Illumination (CIE) proposed a universal method in 1931 to be used in analyzing color. This method distinguishes color into three different tristimulus values. More recently the Munsell system simplifies quantifying color even further through a multidimensional method. L*∗*, a*∗*, b*∗*, h, and C readings can be quantified and compared. L represents the overall lightness of a sample. a*∗* value denotes redness or greenness in a sample, while b*∗* value denotes yellowness and blueness. Hue (h) and Chroma (C) values are derived from a*∗* and b*∗* values with hue expressed in radians or degrees of the angle within the color space and chroma as a measure of intensity as distance from the achromatic center of the color space. Color analysis can indicate surface degradation of product and color difference value (ΔE) can be helpful in distinguishing the difference between storage treatments.

The number generated from this equation can be used as a comparative value against a control sample and utilizes L*∗*, a*∗*, and b*∗* values to express sample color difference. Studies on freezing Atlantic salmon and other varieties of fish have shown similar results with a relationship between freezing rate and color change [7, 17]. Lightness values (L) are seen to increase with freezing rate, while a*∗* and b*∗* values tended to vary [7, 17]. Zhu et al. [7] studied how color difference (ΔE) was affected by freezing rate more so than the freezing, thawing, or cooking methods.

(1) $$\begin{array}{c} \Delta {E^{*}}_{ab}={\left[\;{\left(\;\Delta L^{*}\;\right)}^{2}+{\left(\;\Delta a^{*}\;\right)}^{2}+{\left(\;\Delta b^{*}\;\right)}^{2}\;\right]}^{1/2} \end{array}$$

### 5.3. Salmon Preservation

Freezing is an important method of preservation of Atlantic salmon. Modern refrigeration is often employed in the Atlantic salmon industry and research has revealed that freezing can maintain Atlantic salmon quality at a high level. Indergård et al. [52] tested biochemical, structural, sensory, and microbiological factors of Atlantic salmon after long-term frozen storage. -25°C frozen storage maintained the salmon at an acceptable level after 12 months. -60°C frozen storage reduced drip loss but had no other significant quality improvements than the –25°C frozen. Duun et al. [53] tested the parameters of superchilled Atlantic salmon with vacuum package at -1.4 and -3.6°C. They reported that the shelf-life of vacuum packed salmon fillets was doubled with the utilization of superchilled storage at -1.4°C and -3.6°C. Some processes require thawed Atlantic salmon for preparation to retail markets. Repeated freezing and thawing will inevitably damage salmon fillet tissue. Einen et al. [48] compared how freezing and thawing of fresh or frozen Atlantic salmon fillets affected drip loss, gaping, texture, color, and rigor contraction. They concluded that freezing decreased color quality and firmness and unfrozen fillets had less fillet gaping, higher color score, lower drip loss, and firmer texture. Moreover, as an indispensable step in Atlantic salmon industry, thawing is often implemented more slowly than freezing and usually causes damage to salmon tissue. Although many new freezing and thawing techniques have been utilized in food industry, particular characteristics of Atlantic salmon result in many difficulties in implementing these freezing and thawing techniques. For example, high-pressure thawing (HPT) has been applied in many food marketing aspects. However, Zhu et al. [7] reported that HPT of Atlantic salmon resulted in significant drip loss and structural cracking compared to other thawing methods. Even though HPT significantly accelerated the thawing process, it is not a favorable method for Atlantic salmon thawing because of the quality loss.

There are several challenges and opportunities in the preservation of Atlantic salmon. Damage during repeated freezing and thawing has led to studies to protect stored salmon. Ice-chilling, vacuum, and other fundamental preservation methods are often employed to preserve Atlantic salmon in an attempt to satisfy the rising demand for salmon. Many researchers are studying more efficient ways to preserve Atlantic salmon. Gallart-Jornet et al. [54] showed that superchilling was more effective compared to freezing and ice-packing in preserving raw Atlantic salmon.

### 5.4. Ice Crystallization

**Figure 5:**
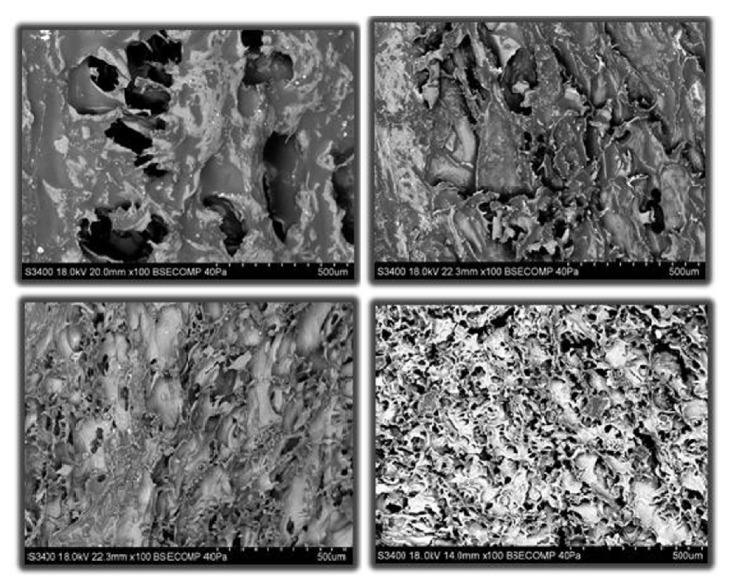
Effect of freezing rate on the microstructure of salmon. Photo micrographs of surface pore. Top left: -7°C(-7°C) corresponds to a treatment frozen at in the -7°C unit to a core temperature of -7°C. Top right: -18°C(-7°C) corresponds to a treatment frozen in the -18°C unit to a core temperature of -7°C. Middle left: -29°C(-7°C) corresponds to a treatment frozen in the -29°C unit to a core temperature of -7°C. Middle right: -106°C(-7°C) corresponds to a treatment frozen in the -77°C to a core temperature of -7°C. (Figure 5 is reproduced from [22] [under the Creative Commons Attribution License/public domain]).

**Figure 6:**
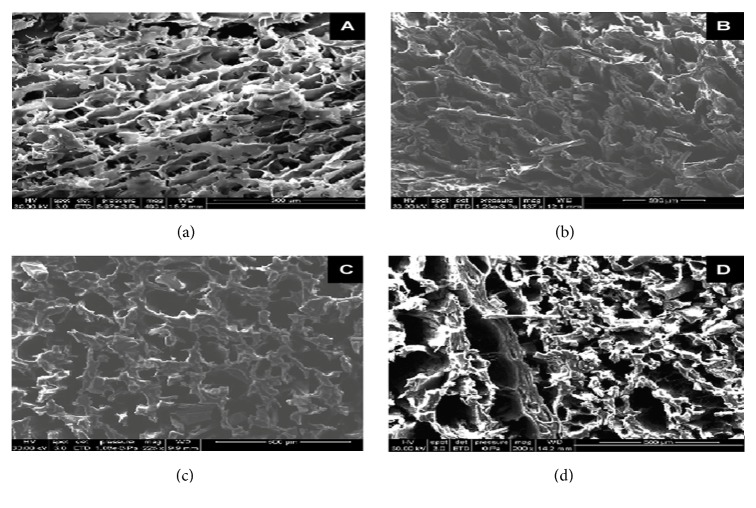
Environmental scanning electron microscopy of freeze-dried salmon. Before freeze-drying, frozen salmon was subjected to state transitions during 4 weeks' storage. (a) Immediately after freezing, (b) T<-27°C(-17°F); (c) -27°C(-17°F) < T < 17°C(1°F); (d) T > 17°C(1°F) (Figure 6 is reproduced from [22] [under the Creative Commons Attribution License/public domain]).

Another effect of freezing foods is ice crystal nucleation and morphology. Ice crystallization and recrystallization affect food structure and food texture. There are three main steps to ice crystallization: (1) nucleation of the crystalline lattice; (2) crystal growth and continuation of ice crystal nucleation; and (3) recrystallization [55]. Freezing rate affects crystal formation and uniformity. Slow freezing leads to larger crystal formation in the extracellular areas of food products, especially within different species of meat and fish. Large crystals as well as small thermodynamically unstable crystals form during the nucleation process and any fluctuation in temperature after freezing causes ice to melt and refreeze, a process also known as recrystallization. Recrystallization during slow freezing causes larger, irregularly spherulite, ice spear-like ice crystals to form. The irregular and extracellular nature of crystals formed from recrystallization damages muscle structure, especially in meat and fish, as connective tissue surfaces deteriorate. Rapid freezing results in ice nucleation within the intracellular areas of food products. These ice crystals are smaller and more uniform, therefore creating a more structurally stable product [4]. For example, different freezing rates affected cell wall structure in salmon (Figure 5). Images “bottom right” and “bottom left” were both subjected to faster freezing methods as compared to images “top right” and “top left”, which were frozen raw and more slowly. Because of different freezing rates, especially shown in image “b”, there is less cell wall damage [4]. Temperature fluctuations and ice recrystallization damage occurs readily in Atlantic salmon muscle structure during four weeks of frozen storage (Figure 6) [13]. Image “a” shows small ice crystals formed uniformly throughout the fish tissue directly after freezing. Immediately after freezing, a sample would not be subjected to recrystallization due to temperature fluctuations, therefore resulting in smaller, more uniform ice crystal pores. Images “b” and “c” represent samples exposed to an increase in their core temperature close to a glassy state and the onset temperature of ice crystals melting. Image “b” has larger ice crystal pores that are still uniform as compared to image “a”. This shows that samples are more structurally stable at a -27°C(-17°F) glassy state. Image “c” shows the ice crystal pores growing larger and varying in uniformity, at a temperature range between -27°C(-17°F) and -17°C(1°F), which allows for ice crystals to melt and recrystallize. Finally, image “d” shows a sample when the temperature surpassed the onset temperature of ice crystal melting with the largest, most irregular ice crystal pores from the whole study. Temperatures above -17°C (1°F) did not provide the most ideal conditions for Atlantic salmon during long-term storage. This study showed the physical damage that can occur to samples frozen to different core temperatures, an area where increased research is needed in the frozen products industry [13]. Thawing allows intracellular water to become permanently extracellular; thus, incurring greater cell wall or muscle damage during freezing will increase water relocation during thawing [56]. Measuring ice crystal pore damage is a good indicator of structural damage imposed by freezing foods. After samples are frozen they can be subjected to freeze-drying, freeze concentration, or free substitution to isolate the pores left behind by ice crystal growth [14].

Freeze-drying has proven to be an effective method to determine ice crystal pore size although it is slower and more expensive than other methods, mainly because ice crystals are vaporized leaving behind a physical pore that emulates the ice crystal's shape [4]. After the pore has been stabilized different techniques can be used to examine pore morphology. Arnaud [57] used optical microscopy to study pore structure and size in ice cream. Fractal, environmental scanning electron microscopy, CT X-ray, and cold stage scanning electron microscopic analysis techniques have been used on different types of fish including Atlantic salmon and sea bass [4, 13, 14]. With these techniques micrograph images can be used to quantify and qualify ice crystal frequency and size. These studies support the objective of the current research, as increasingly more evidence needs to be collected about different freezing rates and how they affect the physical integrity of Atlantic salmon.

### 5.5. Texture

Because of ice crystal growth and recrystallization, another quality parameter that is compromised in fish is texture. Freezing storage temperature affected texture quality in Atlantic salmon fillets more than thawing techniques [17]. Control of pressure and temperature affected ice crystal growth and distribution [17]. Toughness is related to protein and fat content and properties within fish meat. Texture analysis of fish meat differs from other meat types because of variability in muscle structure [21]. There are also added variables such a species, composition, and seasonality that make texture a more challenging attribute to measure. However, trends such as higher resistance and toughness in fish that are in frozen storage for extended periods of time at higher temperatures have been established [16]. Texture changes during frozen storage have also been directly linked to protein denaturation within fish [58]. Salt-water fish, such as Atlantic salmon, may contain higher levels of trimethylamine oxides (TMAO) within red muscle as compared to fresh water fish [59]. TMAO degrades in the presence of TMAase, an enzyme located within fat tissue. The products dimethylamine and formaldehyde are then susceptible to form both intra- and intermolecular crosslinks with protein side chains. The aggregation of these cross linkages causes toughness in fish [17]. Slow freezing could also cause for a tougher sample, as larger ice crystals tend to break down protein structure within fish [42]. Different texture analyses methods include the Kramer Shear Cell method, Warner-Bratzler shear cell method, puncture test, and texture profile analysis (TPA). Each of these methods uses a blade or probe to measure a maximum force for food samples. The Kramer Shear and puncture method can test multiple locations per sample unlike the Warner-Bratzler and TPA methods, which only allow for one or two maximum force readings per sample [16].

### 5.6. Pore Size due to Freezing

The size and number of pores formed during freezing (and refreezing during freezer cycling) affect structural stability within salmon since larger, less uniform pores cause cell wall damage and dehydration in fish muscle fiber structure [60]. Therefore, ice crystal damage may be attributed to the number of nucleated ice crystals first and then to the specific average size of the ice crystals formed [4]. Just as in fish, ice cream storage time and temperature affect ice crystal morphology. Ice crystal formation starts during the churning process and ice crystal size and shape change during storage. A creamy and smooth final ice cream product is associated with a high number of small ice crystals [4, 61]. The lack of statistical difference between average surface pore size in some treatments could be related to the fact that samples frozen more slowly produce large nucleated ice crystals with large variation in size, as well as small thermodynamically unstable ice crystals [4]. Since the food surface freezes more quickly than the center, core ice crystal nucleation and morphology changes occur more slowly than those on the surface. Studies have shown that the differences in ice crystal characteristics on the food surface reflect those seen in the center of food products due to freezing rate [14] but pore number and average size at the center of cylindrical gelatin gels decreased with increasing diameter when frozen at the same freezing rate [14].

### 5.7. Water Holding Capacity (WHC)

Water holding capacity of food products is an important quality parameter due to weight loss during transport and storage, the drip loss during thawing, and the juiciness and tenderness of the meat [62–66]. Water holding capacity is commercially very important due to loss of profit and for consumer acceptance of appearance and texture. Water holding capacity is closely related to textural properties, and a low of WHC is often related to postmortem structural changes in the muscle. Changes include myofilament lattice degradation, denaturation of myosin, and increase of extracellular space [67]. Myofibrils are long rod-like organelles found in cardiac and skeletal muscle comprising nearly 80% of the muscle cell volume [64, 68]. Moreover, almost 85% of the water in a muscle cell is held in the myofibrils [63, 64, 68]. There are three types of water in food which are free, immobilized, and bound. Approximately 90% of water in tissues of fish are immobilized (held by capillary action), mainly in intracellular locations [69, 70]. Free water is easily lost from the tissue and freezing can change immobilized water into free water. Freshly frozen then thawed samples tend to have good water holding, but the ability of the structure to retain immobilized water upon thawing diminishes as length of storage increases due to continued structural changes [71]. Changes associated with water holding capacity have negative outcome on fish texture [72]. NMR relaxation measurements have been used for characterizing changes in water location and water holding capacity during frozen storage of fish [73]. How water molecules react to the imposition and then release (relaxation) of an electric magnetic field are indicative of how tightly they are bond and their location within the meat. The relaxation of an NMR signal in the study of water in meat is reported in terms of two parameters. One parameter, the T1 relaxation, is responsible for the loss of signal intensity while the other parameter, the T2 relaxation, is responsible for the broadening of the signal. The spin-spin (T2) relaxation time changes during frozen storage, from a single peak to a broader multiexponential peak which can be related to texture deterioration [71]. In thawed fish, some of the water that has been isolated from muscle fibers by the formation of ice crystals is not retained or regained by the fibers. The T2 relaxation then shows a long T2 signal peak having a relaxation response similar to that of water [71]. Salmon fillets that were shell/partially frozen using an impingement freezer maintained optimal ice crystal features such as size, distribution, and shape provided that the super chilling is carried rapidly [74]. In addition to physical damage to fibers due to ice crystal formation, an decrease in WHC may result from proteolytic activity in the muscle during storage [75] which causes a loss of water described as a ‘‘leaking out” effect [76].

### 5.8. Protein Stability

Protein denaturation correlates well with textural characteristics. Several researchers have proposed that frozen storage decreases protein quality in fish due to denaturation [77]. Furthermore, protein denaturation during frozen storage causes a decrease in protein solubility due to loss of intermolecular hydrogen or hydrophobic bonds, as well as disulfide bonds and ionic interactions [78–80]. Protein denaturation causes textural changes, especially as a result of changes in the myofibrillar proteins. Protein denaturation is affected by ultimate freezing temperature, storage duration, storage temperature, rigor state at freezing, process of thawing, and temperature fluctuation during storage [4, 81–87]. Protein denaturation reduces the amount of soluble proteins [53, 60]. However, salt-soluble proteins have also been reported to increase during the frozen storage of salmon due to the combined effects of denaturation and proteases to increase extractability [53]. An increase in salt concentration in unfrozen water phase due to the removal of pure water from this phase by freezing leads to myofibril denaturation [88]. Kaale and Eikevik [89] reported no significant difference in water and salt-soluble proteins between surface and center parts of the superchilled salmon samples indicating that rapid freezing minimized the concentration of salts in the unfrozen water phase. However other researchers found that SH groups and the formation of S-S bonds were lost during frozen storage [90–92]. For lizardfish, a consistent decrease in sulfhydryl groups was found throughout storage which was presumed to be caused by formaldehyde formation in this species induced by the oxidation of sulfhydryl groups [93]. Moreover, the masking of sulfhydryl groups by protein aggregates was also presumed to lead to the decrease in free sulfhydryl groups [93]. The S-S formation has been a causative factor for protein aggregation during frozen storage [79, 94, 95]. Changes in proteins could also influence antioxidant activity in the muscle resulting in an increased rate of rancidity during long-term frozen storage. Protein aggregation has been suggested to inhibit the interaction of exogenous hemoglobin with membranes [96]. Therefore, protein aggregation and denaturation occurring through frozen storage may alter the environment of the membranes and could make it more difficult for the antioxidants to interact with oxidative sensitive sites such as phospholipid membranes, thus reducing the efficiency of antioxidants.

### 5.9. Fat Stability and Lipid Oxidation

Storage time and temperature are major factors affecting quality loss and the shelf life of fish [97] with the lipid fraction subject to mainly autoxidative and hydrolytic changes during frozen storage [98]. Several researchers reported that the fat content of fish decreased during frozen storage [99–101]. Arannilewa et al. [101] found that the total lipid content of Tilapia decreased from 9.72% to 7.20% during frozen storage for 60 days [101] primarily due to oxidation resulting in losses in the triglyceride fraction [102, 103]. Similarly, storage at -18°C for 6 months resulted in oxidative changes and decrease in unsaturated fatty acids level in goat meat fat [104]. Peroxidation affects mainly phospholipids, which are in the cell membrane and are the most exposed to attack by free radicals [105]. Other researchers found that the fatty acid of (C16:1) decreased in meat fat during frozen storage [98, 106], while there was no decrease in polyunsaturated fatty acids (PUFA) in frozen meat.

Lipid oxidation is a major factor in the shelf life of fish because it adversely affects the flavor and nutritional value [107]. Fish lipids are rich in long-chain PUFA known for positive health effects; however, they are highly susceptible to oxidation [108]; thus oxidation reduces fish nutritional, texture, and color quality. Many studies showed that the PUFA, especially arachidonic acid (C20:4n-6), eicosanoic acid (EPA), and docosahexaenoic acid (DHA; C22:6n3), decreased as the storage time increased with freezing and refrigeration time [109–111].

Frozen storage reduced the PUFA and increased the saturated fatty acids (SFA) which indicated a substantial loss of nutritional value in the fillets of rainbow trout [110]. The decrease in PUFA might be due to its susceptibility to oxidation; therefore, free PUFA may undergo oxidation as a greater extent than SFA. The first stage of oxidation is the reaction of oxygen with the unsaturated fatty acid molecules creating hydroperoxides which are a primary indicator product of oxidation. Peroxide value (PV) is an early indicator of oxidation (hydroperoxide formation). Peroxide value was lower in cobia frozen at -40 and -80°C compared to -20°C after 6 months of storage [107]. Thiobarbituric acid value which primarily quantifies malondialdehyde is another chemical analysis used as an indicator of lipid oxidation and was found to increase in numerous studies during the storage of fish [107, 112–114]. As expected, a low freezing temperature - 80°C resulted in slower oxidation rate which significantly increased during the storage of anchovies compared to -20°C and -40°C [108]. Lipid oxidation of salmon results in the formation of volatile products such as aldehydes and ketones which are detected by humans as rancid flavors and odors [115]. The rancid off flavor of frozen salmon is primarily because of increase in three aldehydes, (E, Z)-2,6-nonadienal with a cucumber odor, (Z)-3-hexenal with a green odor, and (Z, Z)-3,6-nonadienal with a fatty odor [115, 116], which are formed from the oxidation of n-3 unsaturated fatty acids.

In summary, effects of freezing salmon on quality can be divided into the effects that primarily occur during freezing which are mostly physical effects and effects that occur during frozen storage, which are both physical and chemical/biological. Faster freezing rates form smaller and more numerous ice crystals within salmon, which cause less structural damage and more evenly reflect light. Slower freezing rates form larger and fewer ice crystals, resulting in more structural damage and greater light refraction and a darkening effect of the meat surface. Freshly frozen then thawed samples tend to have good water holding, but the ability of the meat to retain good water holding capacity upon on thawing diminishes the longer the frozen meat is held. Frozen storage factors include physical deterioration due to cycling of temperature which causes water to thaw and refreeze within the meat tissue resulting in the formation of larger ice crystals. Thus, a lower and more consistent holding temperature will slow the formation of large ice crystals and slow the damage to meat tissue. Although lower temperatures slow enzymatic and chemical deterioration, these reactions continue during frozen storage. Browning and autooxidation reactions will proceed during frozen storage to affect flavor, appearance, and nutritional quality of salmon. Salmon is a high quality and highly desirable food and freezing can be a sound method to preserve eating quality in the ever-expanding worldwide market.

## References

[1] USDA (2010). *Dietary Guidelines for Americans*.

[2] Foran J. A., Good D. H., Carpenter D. O., Hamilton M. C., Knuth B. A., Schwager S. J. (2005). Quantitative analysis of the benefits and risks of consuming farmed and wild salmon. *Journal of Nutrition*.

[3] Bell J. G., Henderson R. J., Tocher D. R., Sargent J. R. (2004). Replacement of dietary fish oil with increasing levels of linseed oil: Modification of flesh fatty acid compositions in atlantic salmon (Salmo salar) using a fish oil finishing diet. *Lipids*.

[4] Petzold G., Aguilera J. M. (2009). Ice morphology: Fundamentals and technological applications in foods. *Food Biophysics*.

[5] Johnston W. A., Nicholson F. J., Roge A., Stroud G. D. (1994). Freezing and refrigerated storage in fisheries. *FAO Fisheries Technical Paper*.

[6] Ottestad S., Enersen G., Wold J. P. (2011). Effect of freezing temperature on the color of frozen salmon. *Journal of Food Science*.

[7] Zhu S., Ramaswamy H. S., Simpson B. K. (2004). Effect of high-pressure versus conventional thawing on color, drip loss and texture of Atlantic salmon frozen by different methods. *LWT- Food Science and Technology*.

[8] Strasburg G., Xiong Y. L., Chiang W. (2008). Physiology and chemistry of edible muscle tissues. *Food Chemistry*.

[9] Bahçeci K. S., Serpen A., Gökmen V., Acar J. (2005). Study of lipoxygenase and peroxidase as indicator enzymes in green beans: Change of enzyme activity, ascorbic acid and chlorophylls during frozen storage. *Journal of Food Engineering*.

[10] Eymard S., Carcouët E., Rochet M.-J., Dumay J., Chopin C., Genot C. (2005). Development of lipid oxidation during manufacturing of horse mackerel surimi. *Journal of the Science of Food and Agriculture*.

[11] Nasopoulou C., Psani E., Sioriki E., Demopoulos C. A., Zabetakis I. (2013). Evaluation of sensory and in vitro cardio protective properties of sardine (Sardina pilchardus): The effect of grilling and brining. *Journal of Food and Nutrition Sciences*.

[12] Abdel-Kader Z. M. (1996). Lipid oxidation in chicken as affected by cooking and frozen storage. *Nahrung-Food*.

[13] Syamaladevi R. M., Manahiloh K. N., Muhunthan B., Sablani S. S. (2012). Understanding the influence of state/phase transitions on ice recrystallization in atlantic salmon (salmo salar) during frozen storage. *Food Biophysics*.

[14] Chevalier D., Le Bail A., Ghoul M. (2000). Freezing and ice crystals formed in a cylindrical food model: Part I. Freezing at atmospheric pressure. *Journal of Food Engineering*.

[15] Woinet B., Andrieu J., Laurent M., Min S. G. (1998). Experimental and theoretical study of model food freezing. Part II. Characterization and modelling of the ice crystal size. *Journal of Food Engineering*.

[16] Barroso M., Careche M., Borderías A. J. (1998). Quality control of frozen fish using rheological techniques. *Trends in Food Science & Technology*.

[17] Alizadeh E., Chapleau N., De Lamballerie M., LeBail A. (2007). Effects of freezing and thawing processes on the quality of Atlantic salmon (Salmo salar) fillets. *Journal of Food Science*.

[18] Ayala M. D., López Albors O., Blanco A. (2005). Structural and ultrastructural changes on muscle tissue of sea bass, Dicentrarchus labrax L., after cooking and freezing. *Aquaculture*.

[19] Farouk M. M., Wieliczko K. J., Merts I. (2004). Ultra-fast freezing and low storage temperatures are not necessary to maintain the functional properties of manufacturing beef. *Meat Science*.

[20] Salmon Farming Industry Handbook, Marine Harvest. http://marineharvest.com/product/.

[21] Listrat A., Lebret B., Louveau I. (2016). How muscle structure and composition influence meat and flesh quality. *The Scientific World Journal*.

[22] Remington M. (2017). *The effect of freezing and refrigeration on food quality. Masters Thesis [M.S., thesis]*.

[23] World bank report number 83177-GLB. http://documents.worldbank.org/curated/en/458631468152376668/pdf/831770WP0P11260ES003000Fish0to02030.pdf.

[24] The great salmon run: competition between wild and farmed salmon. http://www.iser.uaa.alaska.edu/people/knapp/personal/pubs/TRAFFIC/The_Great_Salmon_Run.pdf.

[25] Ravichandran S., Kumaravel K., Rameshkumar G., Ajithkumar T. T. (2010). Antimicrobial peptides from the marine fishes. *Research Journal of Immunology*.

[26] Grant W. B. (1997). Dietary links to Alzheimers disease. *Alzheimer's Disease Review*.

[27] Steffens W. (2006). Freshwater fish-wholesome foodstuffs. *Bulgarian Journal of Agricultural Science*.

[28] Love R. M. (1970). *The Chemical Biology of Fishes: With a Key to the Chemical Literature*.

[29] Takama K., Suzuki T., Yoshida K., Arai H., Mitsui T. (1999). Phosphatidylcholine levels and their fatty acid compositions in teleost tissues and squid muscle. *Comparative Biochemistry and Physiology—Part B: Biochemistry & Molecular Biology*.

[30] Varljen J., Šulić S., Brmalj J., Batičić L., Obersnel V., Kapović M. (2003). Lipid classes and fatty acid composition of Diplodus vulgaris and Conger conger originating from the Adriatic Sea. *ood Technology and Biotechnology*.

[31] van Ruth S., Brouwer E., Koot A., Wijtten M. (2014). Seafood and water management. *Foods*.

[32] UK Association of Frozen Food Producers Code of practice on the declaration of fish content in fish products. http://www.seafish.org/media/Publications/Fish_Content_CoP.pdf.

[33] Louka N., Juhel F., Fazilleau V., Loonis P. (2004). A novel colorimetry analysis used to compare different drying fish processes. *Food Control*.

[34] Hassan M. (1996). *Influence of pond fertilization with broiler dropping on the growth performance and meat quality of major carps [Ph.D. thesis]*.

[35] Stien L. H., Kiessling A., Manne F. (2007). Rapid estimation of fat content in salmon fillets by colour image analysis. *Journal of Food Composition and Analysis*.

[36] Fjellanger K., Obach A., Rosenlund G., Kestin S. C., Warris P. D. (2000). Proximate analysis of fish with special emphasis on fat. *Farmed Fish Quality, Fishing News Books*.

[37] Nordøy A., Marchioli R., Arnesen H., Videbæk J. (2001). n-3 polyunsaturated fatty acids and cardiovascular diseases. *Lipids*.

[38] Türkmen A., Türkmen M., Tepe Y., Akyurt I. (2005). Heavy metals in three commercially valuable fish species from İskenderun Bay, Northern East Mediterranean Sea, Turkey. *Food Chemistry*.

[39] Benoit Jr. G. J., Norris E. R. (1944). Studies of Trimethylamine Oxide II. The Origin of Trimethylamine Oxide in Young Salmon. *The Journal of Biological Chemistry*.

[40] Jebsen J. W., Riaz M. (1978). Breakdown products of trimethylamineoxide in air dried stock fish. Means of enhancing the formation of the formaldehyde and dimethylamine. *Fiskeridirektoratets Skrifter Serie Erireing*.

[41] Johnston I. A. (1977). A comparative study of glycolysis in red and white muscles of the trout (Salmo gairdneri) and mirror carp (Cyprinus carpio). *Journal of Fish Biology*.

[42] Santos-Yap E. E. M., Jeremiah L. E. (1996). *Freezing Effects on Food Quality*.

[43] Heldman D. R., Hartel R. W., Heldman., R D. (1998). Freezing and Frozen-Food Storage. *Principles of Food Processing*.

[44] Campañone L. A., Roche L. A., Salvadori V. O., Mascheroni R. H. (2002). Monitoring of Weight Losses in Meat Products during Freezing and Frozen Storage. *Food Science and Technology International*.

[45] Espinoza Rodezno L. A., Sundararajan S., Solval K. M. (2013). Cryogenic and air blast freezing techniques and their effect on the quality of catfish fillets. *LWT- Food Science and Technology*.

[46] Crane D. P., Killourhy C. C., Clapsadl M. D. (2016). Effects of three frozen storage methods on wet weight of fish. *Fisheries Research*.

[47] Turan H., Kaya Y., Erkoyuncu I. (2003). Effects of glazing, packaging and phosphate treatments on drip loss in rainbow trout (Oncorhynchus mykiss) during frozen storage. *Turkish Journal of Fisheries and Aquatic Sciences*.

[48] Einen O., Guerin T., Fjæra S. O., Skjervold P. O. (2002). Freezing of pre-rigor fillets of Atlantic salmon. *Aquaculture*.

[49] Sikorski Z., Olley J., Kostuch S., Olcott H. S. (1976). Protein Changes in Frozen Fish. *C R C Critical Reviews in Food Science and Nutrition*.

[50] Ngapo T. M., Babare I. H., Reynolds J., Mawson R. F. (1999). Freezing and thawing rate effects on drip loss from samples of pork. *Meat Science*.

[51] Pomeranz Y., Meloan C. E., Analysis Food. (1987). Measurement of color. *Food Analysis Theory and Practice*.

[52] Indergård E., Tolstorebrov I., Larsen H., Eikevik T. M. (2014). The influence of long-term storage, temperature and type of packaging materials on the quality characteristics of frozen farmed Atlantic Salmon (*Salmo Salar*). *International Journal of Refrigeration*.

[53] Duun A. S., Rustad T. (2008). Quality of superchilled vacuum packed Atlantic salmon (*Salmo salar*) fillets stored at -1.4 and -3.6°C. *Food Chemistry*.

[54] Gallart-Jornet L., Rustad T., Barat J. M., Fito P., Escriche I. (2007). Effect of superchilled storage on the freshness and salting behaviour of Atlantic salmon (Salmo salar) fillets. *Food Chemistry*.

[55] Hartel R. W. (2002). Crystallization in foods. *Handbook of Industrial Crystallization*.

[56] Sign R. P., Heldman D. R. (2001). *Introduction of Food Engineering*.

[57] Arnaud L., Gay M., Barnola J., Duval P. (1998). Imaging of firn and bubbly ice in coaxial reflected light: a new technique for the characterization of these porous media. *Journal of Glaciology*.

[58] Shenouda S. Y. K. (1980). Theories of protein denaturation during frozen storage of fish flesh. *Advances in Food and Nutrition Research*.

[59] Yamada K. (1967). Occurrence and origin of TMAO in fishes and marine invertebrates. *Bulletin of Japanese Society for the Science of Fish*.

[60] Syamaladevi R. M., Sablani S. S., Tang J., Powers J., Swanson B. G. (2011). Stability of anthocyanins in frozen and freeze-dried raspberries during long-term storage: in relation to glass transition. *Journal of Food Science*.

[61] Hartel R. W. (1996). Ice crystallization during the manufacture of ice cream. *Trends in Food Science & Technology*.

[62] Duun A. S., Rustad T. (2007). Quality changes during superchilled storage of cod (Gadus morhua) fillets. *Food Chemistry*.

[63] den Hertog-Meischke M. J. A., van Laack R. J. L. M., Smulders F. J. M. (1997). The water-holding capacity of fresh meat. *Veterinary Quarterly*.

[64] Huff-Lonergan E. (2002). *Water-holding capacity of fresh. Meat American Meat Science Association*.

[65] Irie M., Izumo A., Mohri S. (1996). Rapid method for determining water-holding capacity in meat using video image analysis and simple formulae. *Meat Science*.

[66] Shaviklo G. R., Thorkelsson G., Arason S. (2010). The influence of additives and frozen storage on functional properties and flow behaviour of fish protein isolated from haddock (melanogrammus aeglefinus). *Turkish Journal of Fisheries and Aquatic Sciences*.

[67] Duun A. S. (2008). *Superchilling of muscle food storage stability and quality aspects of salmon (Salmo salar), cod (Gadus morhua) and pork (Doctoral theses) [Ph.D. thesis]*.

[68] Huff-Lonergan E., Lonergan S. M. (2005). Mechanisms of water-holding capacity of meat: the role of postmortem biochemical and structural changes. *Meat Science*.

[69] Jonsson A., Sigurgisladottir S., Hafsteinsson H., Kristbergsson K. (2001). Textural properties of raw Atlantic salmon (Salmo salar) fillets measured by different methods in comparison to expressible moisture. *Aquaculture Nutrition*.

[70] Offer G., Trinick J. (1983). On the mechanism of water holding in meat: the swelling and shrinking of myofibrils. *Meat Science*.

[71] Medina I., González M. J., Iglesias J., Hedges N. D. (2009). Effect of hydroxycinnamic acids on lipid oxidation and protein changes as well as water holding capacity in frozen minced horse mackerel white muscle. *Food Chemistry*.

[72] Lakshmanan R., Parkinson J. A., Piggott J. R. (2007). High-pressure processing and water-holding capacity of fresh and cold-smoked salmon (Salmo salar). *LWT- Food Science and Technology*.

[73] Brownstein K. R., Tarr C. E. (1979). Importance of classical diffusion in NMR studies of water in biological cells. *Physical Review A: Atomic, Molecular and Optical Physics*.

[74] Kaale L. D., Eikevik T. M., Bardal T., Kjorsvik E., Nordtvedt T. S. (2013). The effect of cooling rates on the ice crystal growth in air-packed salmon fillets during superchilling and superchilled storage. *International Journal of Refrigeration*.

[75] Kaale L. D., Eikevik T. M., Rustad T., Nordtvedt T. S. (2014). Changes in water holding capacity and drip loss of Atlantic salmon (Salmo salar) muscle during superchilled storage. *LWT- Food Science and Technology*.

[76] Olsson G. B., Ofstad R., Lødemel J. B., Olsen R. L. (2003). Changes in water-holding capacity of halibut muscle during cold storage. *LWT—Food Science and Technology*.

[77] MILLS A. (1975). Measuring changes that occur during frozen storage of fish: a review. *International Journal of Food Science & Technology*.

[78] Matsumoto J. J., Whitaker J. R., Fujimoto M. (1980). Chemical deterioration of muscle proteins during frozen storage. *Chemical Deterioration of Proteins*.

[79] Akahane T. (1982). *Freeze denaturation of fish muscle proteins [Ph.D. thesis]*.

[80] Badii F., Howell N. K. (2002). A comparison of biochemical changes in cod (Gadus morhua) and haddock (Melanogrammus aeglefinus) fillets during frozen storage. *Journal of the Science of Food and Agriculture*.

[81] Benjakul S., Visessanguan W., Devahastin S. (2010). Impacts of freezing and frozen storage on quality changes of seafoods. *Physicochemical Aspects of Food Engineering and Processing*.

[82] Blond G., Meste M. L., Murell K. D., Hui Y. H., Nip W.-K., Lim M. H., Lugarreta I. G., Cornillon P. (2004). Principes of frozen storage. *Handbook of Frozen Foods*.

[83] Chevalier D., Sequeira-Munoz A., Bail A. L., Simpson B. K., Ghoul M. (2000). Effect of freezing conditions and storage on ice crystal and drip volume in turbot (Scophthalmus maximus) evaluation of pressure shift freezing vs. air-blast freezing. *Innovative Food Science and Emerging Technologies*.

[84] Hagiwara T., Wang H., Suzuki T., Takai R. (2002). Fractal analysis of ice crystals in frozen food. *Journal of Agricultural and Food Chemistry*.

[85] Jiang S.-T., Lee T.-C. (1985). Changes in Free Amino Acids and Protein Denaturation of Fish Muscle during Frozen Storage. *Journal of Agricultural and Food Chemistry*.

[86] Kiani H., Sun D.-W. (2011). Water crystallization and its importance to freezing of foods: A review. *Trends in Food Science & Technology*.

[87] Mittal G. S., Griffiths M. W., Sun D.-W. (2005). *Pulsed Electric Field Processing of Liquid Foods and Beverage*.

[88] Takahashi K., Inoue N., Shinano H. (1993). Effect of Storage Temperature on Freeze Denaturation of Carp Myofibrils with KC1 or NaCl. *Nippon Suisan Gakkaishi*.

[89] Kaale L. D., Eikevik T. M. (2016). Changes of proteins during superchilled storage of Atlantic salmon muscle (Salmo salar). *Journal of Food Science and Technology*.

[90] LIM H. K., HAARD N. E. (1984). Protein insolubilization in frozen greenland halibut (Reinhardtius hippoglossoides). *Journal of Food Biochemistry*.

[91] LeBlanc E. L., LeBlanc R. J. (1992). Determination of hydrophobicity and reactive groups in proteins of cod (Gadus morhua) muscle during frozen storage. *Food Chemistry*.

[92] Sultanbawa Y., Li-Chan E. C. Y. (2001). Structural changes in natural actomyosin and surimi from ling cod (Ophiodon elongatus) during frozen storage in the absence or presence of cryoprotectants. *Journal of Agricultural and Food Chemistry*.

[93] Benjakul S., Visessanguan W., Thongkaew C., Tanaka M. (2003). Comparative study on physicochemical changes of muscle proteins from some tropical fish during frozen storage. *Food Research International*.

[94] Buttkus H. (1970). Accelerated denaturation of myosin in frozen solution. *Journal of Food Science*.

[95] LeBLANC E. L., LeBLANC R. J. (1989). Separation of Cod (Gadus morhua) Fillet Proteins by Electrophoresis and HPLC after Various Frozen Storage Treatments. *Journal of Food Science*.

[96] Pazos M., Medina I., Hultin H. O. (2005). Effect of pH on hemoglobin-catalyzed lipid oxidation in cod muscle membranes in vitro and in situ. *Journal of Agricultural and Food Chemistry*.

[97] Whittle K. J., Luten J. B., Borrosen T., Oehlenschlager J. (1995). Sea food from producer to consumer, Integrated approach to quality. *Sea Food from Producer to Consumer, Integrated Approach to Quality. Proceedings of The International Seafood Conference on The 25Th Anniversary of WEFTA*.

[98] Zymon M., Strzetelski J., Pustkowiak H., Sosin E. (2007). Effect of freezing and frozen storage on fatty acid profile of calves’ meat. *Polish Journal of Food and Nutrition Sciences*.

[99] Omotosho J. S., Olu O. O. (1995). The effect of food and frozen storage on the nutrient composition of some African fishes.. *Revista de Biología Tropical*.

[100] Kamal M., Islam M. N., Mansur M. A., Hossain M. A., Bhuiyan M. A. I. (1996). Biochemical and sensory evaluation of hilsa fish (Hilsa ilisha) during frozen storage. *Indian Journal of Marine Sciences*.

[101] Arannilewa S. T., Salawu S. O., Sorungbe A. A., Ola-Salawu B. B. (2005). Effect of frozen period on the chemical, microbiological and sensory quality of frozen tilapia fish (*Sarotherodun galiaenus*). *African Journal of Biotechnology*.

[102] Dudakov J. A., Hanash A. M., Jenq R. R. (2012). Interleukin-22 drives endogenous thymic regeneration in mice. *Science*.

[103] Gandotra R. (2012). Change In Proximate Composition And Microbial Count By Low Temperaturepreservation In Fish Muscle Of Labeo Rohita(HamBuch). *IOSR Journal of Pharmacy and Biological Sciences*.

[104] Santos-Filho J. M., Morais S. M., Rondina D., Beserra F., Neiva J. N. M., Magalhães E. F. (2005). Effect of cashew nut supplemented diet, castration, and time of storage on fatty acid composition and cholesterol content of goat meat. *Small Ruminant Research*.

[105] Hultin H. O. (1994). Oxidation of lipids in Seafoods. *Chemistry, Processing Technology and Quality*.

[106] De Pedro E., Murillo M., Salas J., Peña F. Effect of storage time on fatty acid composition of subcutaneous fat.

[107] Taheri S., Motallebi A. A., Fazlara A., Aftabsavar Y., Aubourg S. P. (2012). Influence of vacuum packaging and long term storage on some quality parameters of cobia (Rachycentron canadum) fillets during frozen storage. *American-Eurasian Journal of Agricultural and Environmental Sciences*.

[108] Aydin I., Gokoglu N. (2014). Effects of temperature and time of freezing on lipid oxidation in anchovy (Engraulis encrasicholus) during frozen storage. *European Journal of Lipid Science and Technology*.

[109] Chaijan M., Benjakul S., Visessanguan W., Faustman C. (2006). Changes of lipids in sardine (Sardinella gibbosa) muscle during iced storage. *Food Chemistry*.

[110] Chávez-Mendoza C., García-Macías J. A., Alarcón-Rojo A. D., Ortega-Gutiérrez J. Á., Holguín-Licón C., Corral-Flores G. (2014). Comparison of fatty acid content of fresh and frozen fillets of rainbow trout (Oncorhynchus mykiss) Walbaum. *Brazilian Archives of Biology and Technology*.

[111] Tenyanga N., Womenib H. M., Tiencheub B., Villeneuved P., Lindere M. (2017). Effect of refrigeration time on the lipid oxidation and fatty acid profiles of catfish (Arius maculatus) commercialized in Cameroon. *Grasas Y Aceite*.

[112] Aubourg S. P., Rodríguez A., Gallardo J. M. (2005). Rancidity development during frozen storage of mackerel (Scomber scombrus): Effect of catching season and commercial presentation. *European Journal of Lipid Science and Technology*.

[113] Simeonidou S., Govaris A., Vareltzis K. (1997). Effect of frozen storage on the quality of whole fish and fillets of horse mackerel (Trachurus trachurus) and mediterranean hake (Merluccius mediterraneus). *European Food Research and Technology*.

[114] Sathivel S., Liu Q., Huang J., Prinyawiwatkul W. (2007). The influence of chitosan glazing on the quality of skinless pink salmon (Oncorhynchus gorbuscha) fillets during frozen storage. *Journal of Food Engineering*.

[115] Milo C., Grosch W. (1996). Changes in the Odorants of Boiled Salmon and Cod As Affected by the Storage of the Raw Material. *Journal of Agricultural and Food Chemistry*.

[116] Grosch W. (1987). Reactions of hydroperoxides - Products of low molecular weight. *Autoxidation of Unsaturated Lipids*.

